# Irreversible pulpitis in mature permanent teeth: a cost-effectiveness analysis of pulpotomy versus root canal treatment

**DOI:** 10.1186/s12903-024-04052-9

**Published:** 2024-02-28

**Authors:** Nighat Naved, Fahad Umer, Asif R. Khowaja

**Affiliations:** 1 Operative Dentistry & Endodontics, Aga Khan University Hospital, Karachi, Pakistan; 2 Faculty of Applied Health Sciences, Brock University, St. Catharines, Canada

**Keywords:** Cost-effectiveness analysis, Community healthcare, Cost benefit, Economic evaluation, Endodontics, Pulpotomy, Root canal therapy, Vital pulp therapy (MeSH terms)

## Abstract

**Introduction:**

Evidence-based dentistry suggests pulpotomy as a potential alternative to root canal treatment in mature permanent teeth with irreversible pulpitis. However, the evidence surrounding the cost-valuation and cost-efficacy of this treatment modality is not yet established. In this context, we adopted an economic modeling approach to assess the cost-effectiveness of pulpotomy versus root canal treatment, as this could aid in effective clinical decision-making.

**Methods:**

A Markov model was constructed following a mature permanent tooth with irreversible pulpitis in an 18-year-old patient over a lifetime using TreeAge Pro Healthcare 2022. Transition probabilities were estimated based on existing literature. Costs were estimated based on the United States healthcare following a private-payer perspective and parameter uncertainties were addressed using Monte-Carlo simulations. The model was validated internally by sensitivity analyses, and face validation was performed by an experienced endodontist and health economist.

**Results:**

In the base case scenario, root canal treatment was associated with additional health benefit but at an increased cost (1.08 more years with an incremental cost of 311.20 USD) over a period of an individual’s lifetime. The probabilistic sensitivity analysis revealed pulpotomy to be cost-effective at lower Willingness-To-Pay (WTP) values (99.9% acceptable at 50 USD) whereas increasing the values of WTP threshold root canal treatment was a cost-effective treatment (99.9% acceptable at 550 USD).

**Conclusion:**

Based on current evidence, pulpotomy was a cost-effective treatment option at lower WTP values for the management of irreversible pulpitis in mature permanent teeth. However, by increasing the WTP threshold, root canal treatment became a more cost-effective treatment option over a period of lifetime of an individual.

**Supplementary Information:**

The online version contains supplementary material available at 10.1186/s12903-024-04052-9.

## Introduction

Dental caries is one of the most prevalent diseases with a reported global estimate of 29.4% affecting more than 2.3 billion people worldwide [1, 2]. It can progress to involve the pulp where a part of it may become incapable of self-repair thus, resulting in irreversible pulpitis [3]. The diagnosis, however, may not reflect the actual histological status of the pulp as it is assumed based on clinical symptoms and crude diagnostic tools [4]. Conventionally, irreversible pulpitis has been treated with pulpectomy as it is a predictable approach [5, 6].

Evidence-based histological studies have shown that in teeth with irreversible pulpitis, the microbial invasion is limited to just the coronal portion with the absence of inflammation in the radicular pulp [7]. The emerging insights in pulp biology have led to a better understanding of the disease process [8–10]. In this regard, pulpotomy has emerged as a potential alternative to root canal therapy for the management of irreversible pulpitis in mature permanent teeth [11]. It has the advantage of maintaining pulp vitality, thus retaining its physiological and defensive functions. Secondly, it is a conservative procedure resulting in less weakening of tooth structure [12].

In the routine management of irreversible pulpitis in mature permanent teeth, dentists are faced with the dilemma of deciding between pulpotomy and root canal treatment. Although the latter has been associated with more predictable outcomes, dental practitioners may wish to adopt a biologically driven treatment approach, thereby attempting to maintain pulpal health [11]. Moreover, the costs for both therapies vary, with pulpotomy being less costly initially compared to root canal treatment, however, to the best of our knowledge, the utility of this modality in terms of being cost-effective eventually, in the long run, is yet to be established [13].

When healthcare providers are confronted with a difficult clinical situation, health economics evaluation in the form of a cost-effectiveness analysis can be a pragmatic approach that helps in effective decision-making for the functional allocation of resources [14]. One approach to health economics evaluation is analytic modeling, where the input data can be retrieved from previously published clinical studies [15]. In this regard, we applied the realistic economic model guided by cost and outcome parameters from the relevant literature to evaluate the cost-effectiveness of pulpotomy versus root canal treatment in mature permanent teeth with irreversible pulpitis. Our aim was to test the hypothesis that whether pulpotomy which is less costly at face value was associated with improved health outcomes in terms of life years gained by a tooth over a period of lifetime of an individual.

The operational definition of various terminologies used in this paper related to health economics is presented in Supplementary Table 1.

## Materials and methods

### Study setting and population

The study utilized a Markov simulation model from the private payer (dental insurer) perspective in the context of United States (US) health care using TreeAge Pro Healthcare v2022 (TreeAge Software, Inc, Williamstown, MA). We modeled a cohort of individuals with irreversible pulpitis in mature permanent teeth, receiving any of the two interventions (pulpotomy or root canal treatment). The starting age of the cohort was 18 years, which was then followed over the patient’s lifetime with a remaining life expectancy of 60.6 years according to National Vital Statistics, Center for Disease Control and Prevention (CDC) [16]. The study was reported following the Consolidated Health Economic Evaluation Reporting Standards (CHEERS) guidelines [17].

### Comparator groups

We compared pulpotomy versus root canal treatment in the management of irreversible pulpitis in mature permanent teeth.

### Description of the model

**Fig. 1:**
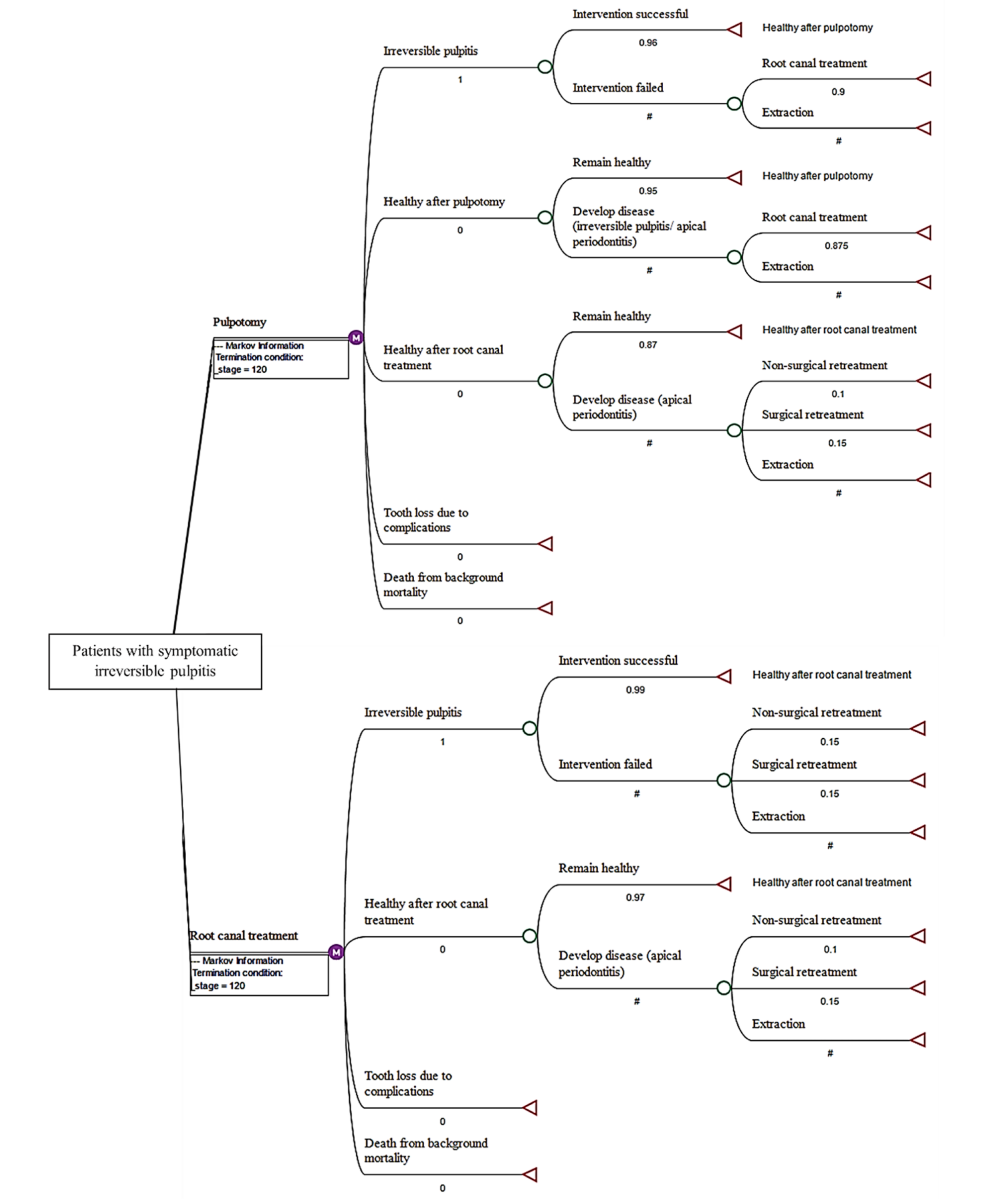
Markov model structure showing starting point (patients with symptomatic irreversible pulpitis), health states, and transitions with probability values assigned. (# refers to 1 minus other probabilities within the same chance node)

The Markov model structure was based on the following key assumptions (Fig. 1):

1. Patients in a state of irreversible pulpitis as the starting point will revert to a healthy state after the successful intervention (either pulpotomy or root canal treatment).
2. Permanent pulpotomy can be performed with success or may lead to persistent symptoms of irreversible pulpitis or pulpal necrosis (loss of vitality) which if left untreated would lead to apical periodontitis.
3. If pulpotomy fails, then either root canal treatment or extraction will be considered.
4. In cases of failed root canal treatment, follow-up treatments would include non-surgical retreatment, surgical retreatment, and/or extraction.

After the intervention was administered, teeth either remained in their respective healthy states or were transferred to the next health states based on the state transition probabilities [18, 19]. We further assumed that after the intervention, teeth were restored with a direct restoration followed by a definitive extra coronal (indirect) restoration, the cost of both was incorporated in the model however, further restorative complications were not simulated. Moreover, the replacement options after extraction as well as periodontal complications were not a part of the analysis.

The simulation was performed in discrete 6-month cycles over a period of the lifetime of an individual. For face validation and to ensure that the components of the model reflect the best available evidence, expert opinion was obtained from an experienced endodontist and a health economist. Moreover, internal validation was performed by varying key parameters in the model in order to check their impact on the results and by performing sensitivity analyses.

### Parameter estimation

A systematic search of the literature was done to estimate the interventions’ success and failure as well as transition probabilities between the health states. The studies that were relevant and reported the outcomes of interest were utilized in the analysis (Supplementary Table 2).

For the success probability of pulpotomy, a mean value was assumed based on the information available from published systematic reviews [11–13, 20]. Likewise, the annual failure rates (AFR) were extracted from literature according to different periods of follow-up after permanent pulpotomy [18]. To estimate the transition probabilities after root canal treatment, data from an existing large-scale study was used [19]. Although not extracted from a systematic review, the data was assumed to be valid and relevant because of the large sample size extrapolated from United States-based setting. To estimate the hazard per six-month cycle for both interventions, cumulative hazard was calculated using AFR which was distributed along cycles assuming a constant hazard per reported period. For follow-up health states, hazards per cycle were calculated using the following formula:

$${h_{(c)}} = {\text{ }}1{\text{ }}--{\text{ }}{\left( {1{\text{ }}--{\text{ }}a{\text{ }} \times {\text{ }}y} \right)^{\left( {1/\left( {2y} \right)} \right)21}}$$

where a is the mean AFR for the respective period y in years.

The costs for both the modalities as well as for the symptomatic management of patients in the diseased state including the charges for the dental visit were simulated using the Dental Fees ADA survey 2020 [21]. The ADA survey represents a robust analysis of self-reported fees by procedure from a nationwide, random sample of dentists and hence can be generalized to a broader population in the US (Table 1). The life expectancy and mortality data of the population were estimated using National Vital Statistics, Center for Disease Control and Prevention (CDC) [16].

**Table 1:** Values for input parameters. *The costs for all the modalities were simulated using the Dental Fees ADA survey 2020

Input parameters	Values
Root canal treatment - **D3330**	1,109.31 USD*
Pulpotomy - **D3220**	210.50 USD*
Direct composite restoration - **D2393**	294.82 USD*
Crown (porcelain fused to metal) - **D2751**	1,095.76 USD*
Nonsurgical retreatment - **D3348**	1,246.06 USD*
Surgical retreatment - **D3425**	961.87 USD*
Extraction - **D7140**	189.83 USD*
Starting age	18 years
Discount rate	0.03
Pulpotomy (success probability) – first year	0.96 [13]
Root canal treatment (success probability) – first year	0.99 [5, 6]
Pulpotomy (success probability) – follow-up years	0.95 [11]
Root canal treatment (success probability) – follow-up years	0.97 [5, 6]
Probability of complications following pulpotomy (follow-up health states)	0.05 [18]
Probability of complications following root canal treatment (follow-up health states)	0.03 [5, 6]
Probability of root canal treatment following failed pulpotomy	0.875 [12, 18]
Probability of extraction following failed pulpotomy	0.125 [18, 20]
Probability of non-surgical retreatment following failed root canal treatment	0.1 [19]
Probability of surgical retreatment following failed root canal treatment	0.15 [19]
Probability of extraction following failed root canal treatment	0.75 [19]

### Cost-effectiveness analysis

Cost-effectiveness was assessed as the incremental cost (USD) relative to the retention time of treated teeth (in years). The base-case incremental cost-effectiveness ratio (ICER) was calculated to check for the dominant treatment modality. We assumed that both the interventions i.e., pulpotomy and root canal treatment were performed according to the standard of care in dentistry. Owing to the US based setting, a discount rate of 3% was applied to the future cost as well as the effectiveness of the interventions [22]. A sensitivity analysis was applied to test robustness by varying the cost of interventions as well as the probability of adverse events. In order to address the parameter uncertainties derived from literature, the probabilistic analysis was applied using Monte Carlo simulations for 10,000 iterations by random sampling of the cost of the interventions as well as the success probabilities derived from literature between 5% and 95% percentiles and the average ICERs were reported. The costs of both interventions (pulpotomy and root canal treatment), as well as follow-up interventions, were allowed to vary in the probabilistic sensitivity analysis with a standard deviation of 10% around mean fixed values (Gamma distribution). Moreover, the success probability for pulpotomy and root canal treatment was allowed to vary by a standard deviation of 0.02 around a fixed input value derived from the literature (Beta distribution) (Supplementary Table 3). Furthermore, in the cost-effectiveness acceptability curve, the probability of a strategy being cost-effective was plotted against different Willingness-To-Pay (WTP) values to predict the cost-effective treatment option.

## Results

### Base-case scenario

In the base-case scenario, an 18-year-old individual with irreversible pulpitis was followed over a lifetime with remaining life expectancy of 60.6 years. At face value, root canal treatment was found to be more costly initially (almost five times) compared to pulpotomy (Table 1). The mean time until teeth were retained in the oral cavity after pulpotomy, and root canal treatment were 15.07 years and 16.15 years respectively. Although root canal treatment was associated with increased health benefits giving 1.08 additional years, it was more costly compared to pulpotomy with an ICER of 288.72 USD/tooth LY gained (Table 2).

**Table 2:** Cost-effectiveness ranking report; pulpotomy versus root canal treatment. Root canal treatment was associated with additional health benefit (1.08 years) at the expense of increased cost (311.20 USD) with an ICER of 288.72 USD/retained life-year of a tooth

Treatment	Cost (USD)	Incremental Cost (USD)	LYs	Incremental LYs	ICER (Incr. Cost/Incr. LYs), USD/LYs
**Pulpotomy**	2469.38	-	15.07	-	-
**Root canal treatment**	2780.59	311.20	16.15	1.08	288.72

### Sensitivity analysis

**Fig. 2:**
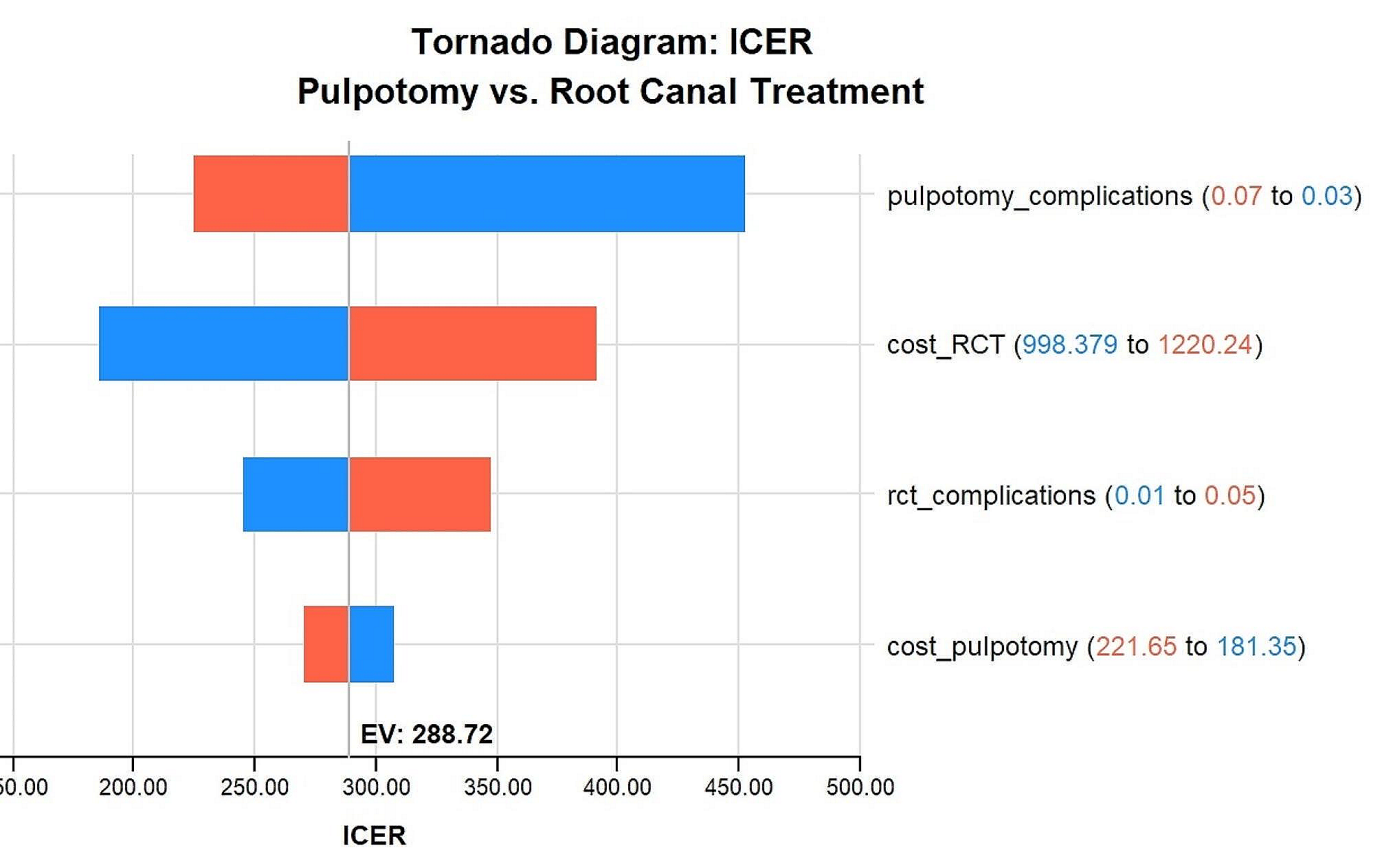
Tornado diagram for sensitivity analysis. Base case ICER was USD 288.72 per tooth LY gained. Note the changes in ICER when the input parameters are slightly changed. Blue color represents the ICER when the values for input parameters were decreased and red color represents the ICER when the values were inflated

A deterministic sensitivity analysis was run using a Tornado diagram for internal validation of the model. The ICER values were found to be altered with the changes in cost of interventions as well as the probability of adverse events showing that our model was sensitive enough to detect changes in the input variables (Fig. 2).

### Probabilistic sensitivity analysis

**Fig. 3:**
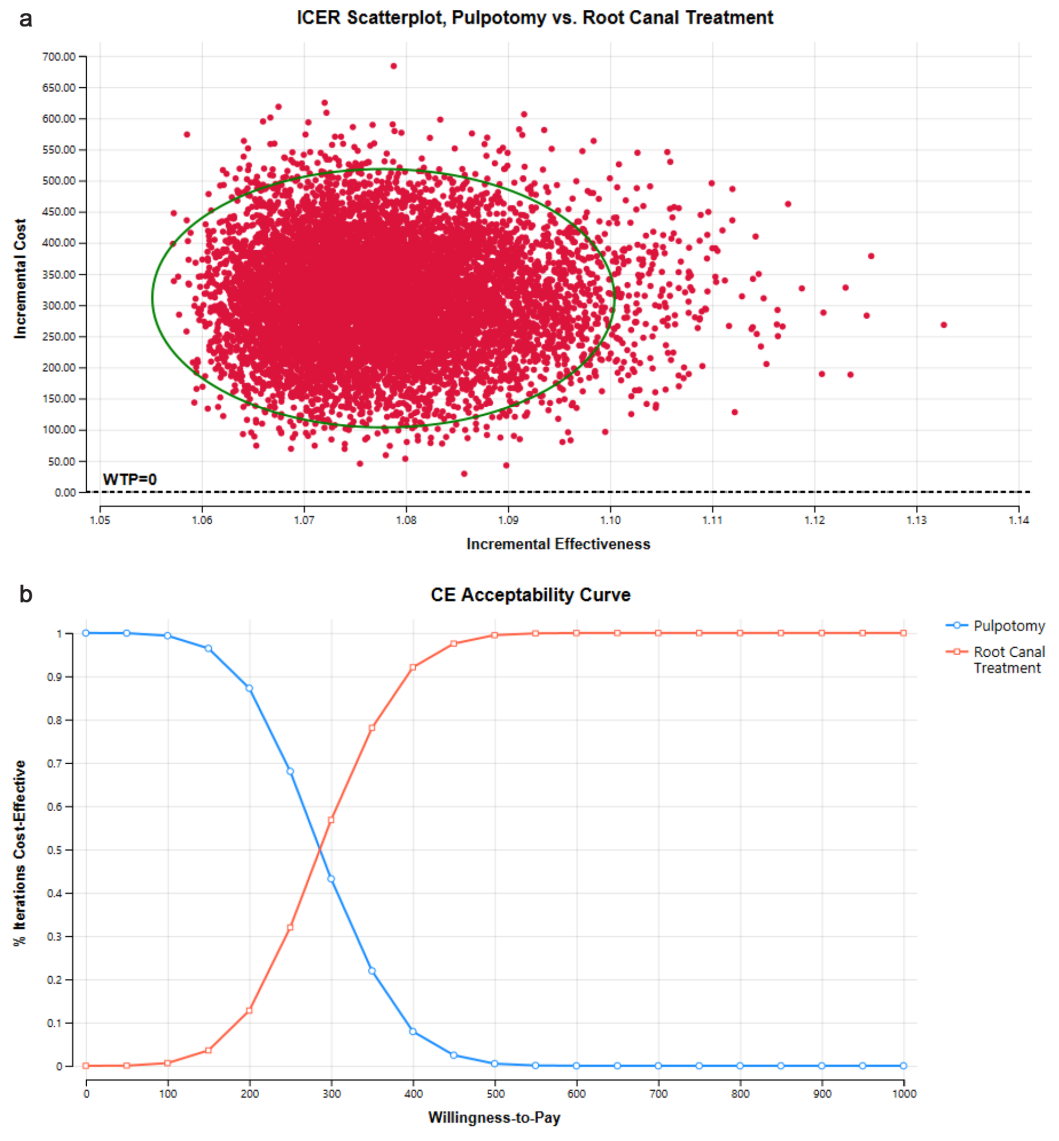
**a** Cost-effectiveness plane. The ellipse represents the ICERs within 95% Credible Intervals. Horizontal and vertical axes show the effectiveness and cost differences between root canal treatment and pulpotomy. The red dots represent scenarios in which ICERs were in the North-East quadrant representing increased cost and increased effectiveness for root canal treatment. **b** Cost-effectiveness acceptability curves; pulpotomy was cost-effective at lower WTP values (99.9% acceptable at 50 USD) whereas by increasing the values of WTP threshold root canal treatment was a cost-effective treatment option (99.9% acceptable at 550 USD)

Using a Monte-Carlo simulation in the probabilistic sensitivity analysis, the model was simulated for 10,000 iterations and the average ICERs were reported within 95% Confidence Intervals. In the analysis, the majority of ICERs were in the North-East quadrant representing increased effectiveness at the expense of increased cost for root canal treatment compared to pulpotomy as represented by red dots (Fig. 3.a).

In the cost-effectiveness acceptability curve, a range of arbitrary WTP values was used for 10,000 iterations since there is no predetermined WTP threshold in dentistry yet. In the analysis, at lower WTP values pulpotomy was an acceptable treatment option in terms of cost-effectiveness (99.9% acceptable at 50 USD), whereas at increased WTP threshold, root canal treatment was an acceptable treatment option (99.9% acceptable at 550 USD) (Fig. 3.b).

## Discussion

Irreversible pulpitis in mature permanent teeth has been routinely managed with root canal treatment as it is reliable, having a success rate of 95–99% [5, 6]. In an era of minimally invasive dentistry, dental practitioners are more inclined towards procedures that preserve the vitality of pulp [23]. Traditionally, pulpotomy was carried out using calcium hydroxide, which has now been superseded by a more biological material like Mineral Trioxide Aggregate (MTA), with a success rate of up to 95% [13, 24]. Despite this high success rate, pulpotomy may fail resulting in necrosis and periapical pathosis [13]. Consequently, teeth may require follow up intervention thereby accruing costs in the long term. Thus, in difficult clinical situations, where dental practitioners might have to choose between two interventions, a cost-effectiveness analysis can play a pivotal role as this aids in effective decision making allowing for the functional allocation of resources.

Cost-utility analysis and cost-effectiveness analysis are the two common types of economic evaluation used to evaluate the cost-effectiveness of a certain intervention. Cost-utility analysis reports the qualitative as well quantitative outcomes in the form of a single measure such as Quality Adjusted Life Years (QALYs). From our perspective it may not be an appropriate tool as it considers the utility values derived from various healthcare indices that do not detect the true impact of oral conditions [25]. For instance, the most widely used index is the EuroQol (EQ-5D-5 L) tool that evaluates five different attributes (mobility, self-care, usual activities, pain/discomfort, anxiety/depression) associated with the quality of life of an individual [26]. This concept was further modified, and Quality Adjusted Tooth Years (QATYs) and Quality Adjusted Prostheses Years (QAPYs) were introduced however, they have not developed much over the years [27].

Cost-effectiveness analysis has been extensively studied in dentistry and is the widely used tool to assess the cost-effectiveness of certain intervention in the context of a specific healthcare setting [28]. It compares the cost and effectiveness of different interventions based on a shared outcome measure (such as retention of tooth or reduction in DMFT index etc.) [29]. In this regard, we developed a Markov simulation model from the private payer perspective in the context of United States (US) health care to follow mature permanent teeth with irreversible pulpitis receiving either pulpotomy or root canal therapy, based on the best available evidence.

The findings of our study suggested that root canal treatment was associated with marginally increased health benefits in terms of number of years a tooth was retained in the oral cavity (1.08 more years) and at the expense of increased cost. In contrast, pulpotomy was associated with reduced health benefits as well as reduced cost. Moreover, to maximize the yield of the model, a lifetime horizon was chosen. This was possible with the incorporation of the function of hazards per cycle that takes into account the adverse events over the longest follow-up period available from literature and then predicts the incidence of adverse events over the entire life course [30].

Some previous studies in dentistry have utilized a similar model to evaluate the cost-effectiveness of direct pulp capping compared to root canal treatment following vital pulp exposures [31, 32]. A study conducted on the Scandinavian population concluded that direct pulp capping was more cost-effective compared to root canal treatment in children and adolescents [31]. Likewise, a study on German population reported direct pulp capping to be more effective in younger patients and for occlusal exposure sites whereas root canal treatment was more cost-effective in older patients and teeth with proximal exposures [32]. However, in our study the effect of exposure site as well as the influence of age factor was not analyzed.

Given that the findings are in the context of United States healthcare, one may have concern that our results are not adequately generalizable. This is the inherent limitation of a cost-effectiveness analysis as they could only be as good as the data in the published studies which may be biased in some way or the other. However, to address the uncertainties in model inputs and to quantify the level of confidence in the output, a probabilistic sensitivity analysis was used. Therefore, considering the robustness of our model, the outcome is unlikely to be affected under different testing conditions.

The results of the probabilistic sensitivity analysis revealed the majority of ICERs in the North-East quadrant representing increased cost and increased effectiveness for root canal treatment compared to pulpotomy. This is where a trade-off comes into play in such cases, and it is the Willingness-to-Pay (WTP) threshold that decides whether the intervention is cost-effective or not. Moreover, in the US a WTP threshold of USD 50,000 per Life Year gained for an individual is referenced by the researchers however, there is no established consensus on the WTP threshold value for a tooth’s LY gained. Hence in our analysis, the probability of the cost-effectiveness of pulpotomy and root canal treatment was plotted against a range of arbitrary WTP values to determine their acceptability at different values. At lower WTP values, pulpotomy turned out to be a cost-effective treatment option whereas at increased WTP threshold values, root canal treatment was more cost-effective. Owing to the comparable effectiveness of both interventions in terms of life years gained by a tooth, this has considerable implications for healthcare professionals as well as policy makers particularly in areas where there are financial constraints, enabling them to choose between pulpotomy and root canal treatment in the management of irreversible pulpitis in mature permanent teeth.

Having said that, our analysis had certain limitations. Owing to the lack of primary data, the opportunity cost of patients’ time in treatment as well as that of the healthcare providers was not taken into consideration. Moreover, the effect of etiological factors (i.e., caries, trauma, or any developmental anomaly), the exposure site (i.e., occlusal, proximal, etc.) as well as patient related factors on the success probability of individual modalities, was not accounted for. Lastly, when pulpotomy fails and root canal treatment is performed, there is a risk of canal calcification. Since these risks and potential complications of future root canal treatment have not been evaluated in endodontic literature, therefore it was not considered in this study.

## Conclusion

In the context of United States healthcare, pulpotomy was an acceptable treatment option in terms of cost-effectiveness at lower WTP values for the management of irreversible pulpitis in mature permanent teeth. However, by increasing the WTP threshold, root canal treatment became a more acceptable treatment option over a period of lifetime of an individual.

### Future directions

Applying a societal perspective includes a broad spectrum of public benefits, however, restricting it to the insurer perspective was primarily due to the lack of primary data surrounding the indirect costs involved. Therefore, it is an important limitation of our study, and the authors recommend future research on identifying cost drivers necessary to measure the indirect costs in this area. Moreover, since there is scarce evidence regarding the WTP threshold for a tooth’s LY gained, future research work should also be directed to explore this undiscovered domain.

### Electronic supplementary material

Below is the link to the electronic supplementary material.

Supplementary Material 1

## Data Availability

The datasets generated or analyzed during this study are available from the corresponding author upon reasonable request.
