# Supplementary material for: Irreversible pulpitis in mature permanent teeth: a cost-effectiveness analysis of pulpotomy versus root canal treatment
Source: BMC Oral Health. 2024 Feb 28;24:285. doi: 10.1186/s12903-024-04052-9 (PMC10902936; doi:10.1186/s12903-024-04052-9)
Supplement: Supplementary file 1 — Supplementary Material 1 [file 12903_2024_4052_MOESM1_ESM.docx]

| **Economic evaluation:** Economic evaluation analyzes the costs and effects of alternative interventions in order to support decision-making.^1^  They are of four basic types, which vary in terms of outcome with the cost being a common aspect (cost-benefit analysis, cost-utility analysis, cost-minimization analysis, and cost-effectiveness analysis).^2^ |
| --- |
| **Cost-benefit analysis:** Cost-benefit analysis (CBA) compares the consequences of interventions in which both the costs and outcomes are expressed in monetary terms.^3^ It covers the broader impact of intervention on the economy as a whole. |
| **Cost-utility analysis:** Cost-utility analysis (CUA) measures health effects in terms of both quantity (life years) and quality of life which are combined into a single measure of health: quality-adjusted life years (QALYs).^4^ |
| **Cost-minimization analysis:** Cost minimization analysis compares the costs of alternative interventions that are assumed to have an equivalent effectiveness.^5^ |
| **Cost-effectiveness analysis:** Cost-effectiveness analysis compares an intervention to another by estimating how much it costs to gain a unit of health outcome, like a life year gained or death prevented.^6^ |
| **Incremental cost-effectiveness ratio (ICER):** An incremental cost-effectiveness ratio (ICER) is the main output of an economic evaluation and is calculated by dividing the difference in total costs by the difference in the chosen measure of health outcomes like Life Years (LYs) gained etc.^7^  ICERs may be positive or negative. Positive ICER values indicate the additional cost per effectiveness gain, whereas negative values indicate the additional cost per decreased effectiveness. Strategies with positive ICERs dominate the comparators whereas that with negative ICERs are dominated by the comparator. |
| **Willingness-to-pay (WTP):** Willingness-to-pay (WTP) is an estimate of what a consumer of the health care system might be prepared to pay for the health benefit and is often based on a country’s per capita gross domestic product.^8^ In the United States, a WTP threshold of $50,000 to $100,000 is referenced and used by public and private policymakers, insurers, and researchers. |
| **Base case analysis:** A base case analysis refers to the results obtained from running an economic model with the preferred set of assumptions and input values.^9^ |
| **Probabilistic Sensitivity Analysis (PSA):** Probabilistic sensitivity analysis (PSA) allows quantifying the level of confidence about uncertainty in the model inputs which may have been derived from clinical trials or expert opinion. The parameters are represented as distributions around the point estimate, which can be summarized using mean and standard deviation for a normal distribution.^10^ |

**Supplementary Table 1** Operational definition of terminologies related to health economics

| **Literature** | **Source** |
| --- | --- |
| 1. Pulpotomy for mature carious teeth with symptoms of irreversible pulpitis: A systematic review. | Cushley S, Duncan HF, Lappin MJ, Tomson PL, Lundy FT, Cooper P, Clarke M, El Karim IA.  **(J Dent. 2019)** ^14^ |
| 1. Coronal pulpotomy for cariously exposed permanent posterior teeth with closed apices: A systematic review and meta-analysis. | Alqaderi H, Lee CT, Borzangy S, Pagonis TC.  **(J Dent. 2016)** ^15^ |
| 1. Pulpotomy for carious pulp exposures in permanent teeth: A systematic review and meta-analysis. | Li Y, Sui B, Dahl C, Bergeron B, Shipman P, Niu L, Chen J, Tay FR.  **(J Dent. 2019)** ^16^ |
| 1. Vital pulp therapy in carious pulp-exposed permanent teeth: an umbrella review. | Leong DJX, Yap AU.  **(Clin Oral Investig. 2021)** ^17^ |
| 1. Long-term Pulpal and Restorative Outcomes of Pulpotomy in Mature Permanent Teeth. | Tan SY, Yu VSH, Lim KC, Tan BCK, Neo CLJ, Shen L, Messer HH.  **(J Endod. 2020)** ^18^ |
| 1. Outcome of primary root canal treatment: systematic review of the literature - part 1. Effects of study characteristics on probability of success. | Ng YL, Mann V, Rahbaran S, Lewsey J, Gulabivala K  **(Int Endod J. 2007)** ^11^ |
| 1. Outcome of primary root canal treatment: systematic review of the literature -- Part 2. Influence of clinical factors. | Ng YL, Mann V, Rahbaran S, Lewsey J, Gulabivala K.  **(Int Endod J. 2008)** ^12^ |
| 1. Endodontic treatment outcomes in a large patient population in the USA: an epidemiological study. | Salehrabi R, Rotstein I.  **(J Endod. 2004)** ^13^ |

**Supplementary Table 2** An extensive literature search was done, and the above-mentioned studies were utilized to estimate the success of the interventions as well as transition probabilities.

| **Input parameters** | **Mean ± SD values** |
| --- | --- |
| Pulpotomy | 210.50 ± 10% |
| Root canal treatment | 1,109.31 ± 10% |
| Direct composite restoration | 294.82 ± 10% |
| Crown (porcelain fused to metal) | 1,095.76 ± 10% |
| Nonsurgical retreatment | 1,246.06 ± 10% |
| Surgical retreatment | 961.87 ± 10% |
| Extraction | 189.83 ± 10% |
| Success probability of pulpotomy | 0.05 ± 0.02 |
| Success probability of root canal treatment | 0.03 ± 0.02 |

**Link to the model file:**

**Supplementary Table 3:** Mean and SD values for input parameters

[**https://drive.google.com/file/d/1qjnD6LmhGRSDnoEcxdwvGa6M0i6w4Ghx/view?usp=sharing**](https://drive.google.com/file/d/1qjnD6LmhGRSDnoEcxdwvGa6M0i6w4Ghx/view?usp=sharing)

**References:**

1. [Available from: <https://yhec.co.uk/glossary/economic-evaluation>.] [cited 2023 Dec 7]

2. Rogers HJ, Freitas RD, Beeson MJ, Vernazza CR. Economic evaluations in paediatric dentistry clinical trials. Int J Paediatr Dent. 2020;31 Suppl 1:56-65.

3. [Available from: <https://yhec.co.uk/glossary/cost-benefit-analysis>.] [cited 2023 Dec 7]

4. [Available from: <https://www.gov.uk/guidance/cost-utility-analysis-health-economic-studies>.] [cited 2023 Dec 7]

5. [Available from: <https://yhec.co.uk/glossary/cost-minimisation-analysis>.] [cited 2023 Dec 7]

6. CEA. [Available from: <https://www.cdc.gov/policy/polaris/economics/cost-effectiveness/index.html>.] [cited 2023 Dec 7]

7. ICER. [Available from: <https://yhec.co.uk/glossary/incremental-cost-effectiveness-ratio-icer/>.] [cited 2023 Dec 7]

8. WTP. [Available from: <https://yhec.co.uk/glossary/willingness-to-pay/>.] [cited 2023 Dec 7]

9. BCA. [Available from: <https://yhec.co.uk/glossary/base-case-analysis/>.] [cited 2023 Dec 7]

10. PSA. [Available from: <https://yhec.co.uk/glossary/probabilistic-stochastic-sensitivity-analysis/>.] [cited 2023 Dec 7]

11. Ng YL, Mann V, Rahbaran S, Lewsey J, Gulabivala K. Outcome of primary root canal treatment: systematic review of the literature - part 1. Effects of study characteristics on probability of success. Int Endod J. 2007;40(12):921-39.

12. Ng YL, Mann V, Rahbaran S, Lewsey J, Gulabivala K. Outcome of primary root canal treatment: systematic review of the literature -- Part 2. Influence of clinical factors. Int Endod J. 2008;41(1):6-31.

13. Salehrabi R, Rotstein I. Endodontic treatment outcomes in a large patient population in the USA: an epidemiological study. J. Endod. 2004;30(12):846-50.

14. Cushley S, Duncan HF, Lappin MJ, Tomson PL, Lundy FT, Cooper P, et al. Pulpotomy for mature carious teeth with symptoms of irreversible pulpitis: A systematic review. J Dent. 2019;88:103158.

15. Alqaderi H, Lee CT, Borzangy S, Pagonis TC. Coronal pulpotomy for cariously exposed permanent posterior teeth with closed apices: A systematic review and meta-analysis. J Dent. 2016;44:1-7.

16. Li Y, Sui B, Dahl C, Bergeron B, Shipman P, Niu L, et al. Pulpotomy for carious pulp exposures in permanent teeth: A systematic review and meta-analysis. J Dent. 2019;84:1-8.

17. Leong DJX, Yap AU. Vital pulp therapy in carious pulp-exposed permanent teeth: an umbrella review. Clin Oral Investig. 2021;25(12):6743-56.

18. Tan SY, Yu VSH, Lim KC, Tan BCK, Neo CLJ, Shen L, et al. Long-term Pulpal and Restorative Outcomes of Pulpotomy in Mature Permanent Teeth. J. Endod. 2020;46(3):383-90.
